# Is walking to school associated with improved metabolic health?

**DOI:** 10.1186/1479-5868-10-12

**Published:** 2013-01-29

**Authors:** Andreia Nogueira Pizarro, José Carlos Ribeiro, Elisa Amélia Marques, Jorge Mota, Maria Paula Santos

**Affiliations:** 1 Research Centre in Physical Activity, Health and Leisure, Faculty of Sports, University of Porto, Rua Dr. Plácido Costa, 91, 4200-450, Porto, Portugal; 2 Higher Education Institute of Maia (ISMAI), Maia, Portugal

**Keywords:** HDL-C, Waist circumference, Active commuting, Cardiovascular disease risk factors

## Abstract

**Background:**

Active commuting to/from school is an important source of physical activity that has been declining over the past years. Although it is an affordable and simple way of increasing physical activity levels it is still unclear whether it has enough potential to improve health. Therefore, the aim of this cross sectional study was to examine the relationship between active commuting to/from school and metabolic risk factors in 10 to 12 year old children.

**Methods:**

Participants were 229 adolescents, selected through consecutive sampling, (121 girls) with mean age of 11.65 (±0.73) years old from Porto, Portugal. Means of transport to/from school was accessed by asking: ”How do you usually travel to school?” and “How do you usually travel from school?”. Active commuting was considered if children reported at least one of the trips (to or from school) by active means. Total physical activity was obtained with Actigraph accelerometer for 7 consecutive days. Lipid profile measurements were conducted with Cholestech LDX*®* analyser. Waist circumference and blood pressure were measured by standard methods. The criteria for metabolic syndrome defined by International Diabetes Federation for children and adolescents were used.

**Results:**

Adjusted binary logistic regression analysis suggested that walkers have higher odds to have a better waist circumference (OR = 2.64, 95% CI = 1.63-6.01) and better high density lipoprotein cholesterol (OR = 2.14, 95% CI = 1.01-4.52) profiles than non-active commuters, independent of moderate-to-vigorous physical activity. No associations were found for other metabolic risk factors.

**Conclusions:**

Exertions to increase and maintain walking to school may be particularly relevant as it is likely to have a positive impact on children’s health and eventually decrease metabolic and cardiovascular diseases.

## Introduction

Being physically active can help reduce the prevalence of obesity in children and is associated with a decrease in cardiovascular risk factors such as lipid disorders, high blood pressure, insulin resistance among others [1]. Recent studies suggest that low levels of high-density lipoprotein cholesterol (HDL-C) are an important and independent risk factor for cardiovascular disease (CVD) [2] and are linked to worse CVD outcomes [3]. This should be a major cause of concern because cardiovascular risk factors tend to track into adulthood [4] and the process of atherosclerosis, the pathologic basis for clinical CVD starts early in childhood [1].

Active commuting to/from school (ACS) should be consider a key factor to reduce important negative health outcomes as it has been identified as an important source of physical activity (PA) for young people [5,6]. However in Portugal, similarly to most developed countries, data on transport suggests a decline in active commuting over the past years. For example, in Porto, walking trips to work/school decreased from 27% in 1991 to only 19% in 2001. Inversely, car was the most used transport to work/school in 2001 (49%), increasing over than 100% when compared to 1991 when only 23% of the journeys were made by car, showing dramatically changes in transport patterns (INE, 2003). These shifts are replicated, all over the world, in children’s decreasing number of walking trips to/from school [7,8].

Although some evidence supports that ACS is associated with a healthier body composition and higher levels of cardiorespiratory fitness among youth [9], it remains unclear whether ACS has the potential to improve health through a better metabolic profile as research on this topic is scarce. To our knowledge, only two studies have addressed the relationship between ACS and metabolic risk factors in children [10,11]. A research in Danish children and adolescents found no differences in CVD risk factors between passive travelers and walkers, but 15-year-olds that cycled to school showed consistently better CVD risk profile compared with noncyclists [10]. Moreover children who did not cycle at baseline and had changed to cycling at follow up had better cholesterol/HDL ratio, better glucose metabolism, and a lower composite CVD risk factor score than those who did not cycle at either time point. On the other hand, Chillón and colleagues [11] found no significant associations between means of transport to school and cardio metabolic risk factors in a longitudinal study in Swedish children. Although both studies were developed in north European countries, with strong traditions of cycling to school, findings show conflicting results. In addition, PA in these studies could be underestimated as bicycling is not accurately measured by the accelerometer, therefore influencing the results. Moreover, any of the studies considered diet intake although it has a well-recognized role affecting cardiovascular health and risk factors for CVD [12]. The study from Andersen and coworkers [10] found no differences between walking and non-active means of transport in relation to CVD risk profile, however previous research show that walking has the potential to play a key role in the primary and secondary prevention of CVD in younger, middle, and older men and women, in both healthy and patient populations [13].

The aim of this study was to examine the relationship between ACS and metabolic risk factors having known potential confounders in consideration. It is our intention to improve the understanding of this issue since ACS is likely to be easily incorporated into daily living activities, positively impacting health outcomes.

## Methods

### Participants

Data for this study is a part of SALTA PROJECT STUDY (Environmental Support for Leisure and Active Transport), a longitudinal study being developed in Porto area, Portugal, designed to examine environmental and social influences on PA. Ethical approval for this study was obtained from the Faculty of Sports ethics committee, the Foundation for the Science and Technology and by the regional section of the ministry of Education.

All public middle-schools in Porto area were invited to take part in the SALTA PROJECT by letter, email and telephone. From a total of 65 schools, 37 declined to participate, 13 did not reply to our invitation and 15 schools agreed to take part in this study. Due to lack of resources and failure in bilateral talks, data were only collected in 9 middle-schools with a total population of 1555 students in the 6^th^ grade. Respondents included 652 children who agreed to participate and had parental written consent to take part in the study. After eliminating subjects due to collecting errors, SALTA PROJECT final sample comprised 641 children (41.2%; 342 boys and 299 girls) with a mean age of 11.60 years old. From those, 121 girls and 108 boys with mean age of 11.65 (±0.73) years old had parental authorization to collect capillary blood samples and took part in this particular study.

### Testing protocol

Data collection was performed in the school gymnasium between 08:20 and 11:00 a.m. As children were overnight fasting, lipid profile was firstly accessed. After blood samples were taken children were given light breakfast. Subsequently, participants fulfilled a demographic questionnaire and determined their maturational stage. Following a 5 minute rest in a separate room, blood pressure was measured. Afterwards anthropometric data was collected and nutritional information was gathered; lastly accelerometer devices were placed right before the testing protocol was over.

### Measurements

#### Means of transport to and from school

Means of transport to and from school was accessed by questionnaire. Participants were asked “How do you usually travel to school?” and “How do you usually travel from school?”. Possible answers included walking, cycling, by car or by public transport. Based on their answers the respondents were categorized as active commuters (walking and cycling) or non-active commuters (car or public transport) to/from school. If subjects reported at least one of the trips as active they were included in the active commuting group [14]. Previous studies have demonstrated evidence for reliability and validity for similar questions [15].

#### Metabolic risk factors

*Waist circumference* (WC) was measured with a non-metallic tape midway between the lower rib margin to the anterior superior iliac crest [16] at the end of normal expiration.

*Capillary fasting blood samples* were taken from the middle finger by trained professionals according to Center of Disease Control capillary blood sample protocol. Blood samples were drawn in capillary tubes (35 μl, Selzer) coated with lithium heparin and immediately assayed using Colestech LDX Analyser in order to obtain values of plasmatic total cholesterol (TC), HDL-C, triglycerides (TG), and fasting glucose (GLU). A daily optic check was conducted on the analyzer used for the study.

*Systolic and diastolic blood pressure* measurements were performed with Colin model BP 8800 (Critikron, Inc., Tampa, FL) on the right arm after a 5 minute rest. Participants were in a comfortably seated position with their back supported and legs not crossed. The arm was bared without constrictive clothes, supported at the heart level and resting in a table. At least two readings were performed at 1-minute intervals; if there was a > 5 mmHg difference between the first and second readings additional readings were done. The average of two readings with < 5 mmHg difference were used for analysis [17].

#### Clustered metabolic risk score

The cluster for metabolic syndrome for children and adolescents defined by International Diabetes Federation (IDF) was used to access the metabolic risk factors [18] and included WC ≥ 90 ^th^ percentile, HDL-C <40 mg/dL, TG ≥ 150 mg/dL, GLU ≥ 100 mg/dL, and systolic blood pressure ≥ 130 or diastolic blood pressure ≥ 85 mmHg. In regards to blood pressure if the participant had at least one of the systolic or diastolic blood pressure values above the reference they were included in the risk group.

#### Confounders

*Total physical activity* was measured with Actigraph accelerometers, model GT1M (Actigraph, Pensacola, FL). Participants were instructed to use the accelerometer attached to an elastic belt and placed above the right iliac crest for 7 consecutive days. Instructions were given to wear the monitor all times except when sleeping, bathing, swimming or other water activities. Data was collected in 30 seconds epochs and a minimum recording of 8 hours on at least 4 days (1 weekend day) were considered valid data. 60 minutes of consecutive zeros were considered invalid data. PA data was processed using Actilife software (Actigraph LLC Pensacola, FL) and summarized as time spent in moderate and vigorous PA (MVPA), defined as ≥ 2296 counts/min. This cut points for PA intensities developed by Evenson and colleagues [19] seem to exhibit significantly better accuracy than others in children and adolescents [20].

*Anthropometric measurements* were taken with children in bare feet and lightly dressed. Height (cm) was measured with SECA 206 Bodymeter Measuring Tape (SECA, Hamburg Germany). Weight (Kg) and body fat percentage were accessed using digital scales (TANITA BF-522 W, Tokyo, Japan). Body mass index (BMI) was calculated as weight (kg) / height (m)^2^. Overweight and obesity were defined according to sex- and age-specific BMI cut points established by Cole et al. [21].

*Socioeconomic status* (SES) was obtained based on parents report of their educational level and was collapsed into three categories according to Portuguese Educational System: ≤ 6 years (low), 7–12 years (medium) and > 12 (high) similarly to procedures applied in the Portuguese context before [22].

Participants were asked to self-determine their *pubertal stage* using the pattern of development diagrams for pubic hair and breasts or genital development established by Tanner [23]. If the responses differed between pubic hair and breasts/genital development, then the pubic hair stage was used.

*Food intake* was assessed by a trained nutritionist using the 24-hour recall and supported by a food quantification manual to allow more accurate portion size estimation. Data were then analyzed using Food Processor Plus (ESHA Research, Salem, OR). The 24-h recall is the most commonly used dietary assessment method because it is easy to administer, can be performed in large-scale studies [24,25] and can be used to assess adequacy of energy and macronutrient intakes.

### Statistical analyses

Data was analysed using IBM SPSS Statistics (version 20; SPSS, Inc., Chicago, IL). Significance level was set at 5% (p < 0.05). Descriptive statistics were used to characterize the sample. Point biserial correlation determined the associations between walking and MVPA. Regression analysis models were conducted to establish associations between metabolic risk factors and walking. Known potential confounders for the CVD risk factors, namely body fat percentage, calories from fat, age, gender, SES, pubertal stage and minutes in MVPA were included in the analysis to find the model that best fits the observed data. Variables were retained in the models if backward elimination resulted in a greater than 10% change in the estimated effect measures. Interactions between walking and each confounder were studied when accessing the associations of walking and each metabolic risk factor.

## Results

Demographic characteristics of the initial sample did not differ significantly from the final sample included in this particular study.

Sample characteristics are presented in Table 1. About 53% of the students were girls and most prevalent tanner stages were 3 and 4. Sample SES was predominantly (87%) medium and low. Among 229 students 75% made at least one trip (to or from school) by active means. Interestingly, walking was the only active means of transport reported. Noteworthy 85.5% of the children did not achieve daily PA recommendations of 60 minutes of MVPA and MVPA mean time was about 40 minutes. Association between walking and MVPA was also assessed but no significant relation was found (p = 0.102). Mean sample metabolic components were within normal values [18]. Table 2 provides the number of subjects exhibiting each component of the metabolic syndrome. Metabolic syndrome defined by abdominal obesity (WC ≥ 90^th^ percentile) and the presence of two or more other clinical features (HDL <40 mg/dL, TG ≥150 mg/dL, GLU ≥ 100 mg/dL and systolic blood pressure ≥ 130 or diastolic blood pressure ≥ 85 mmHg) was observed in only 3% of the sample. Most prevalent lipid disorder was low levels of HDL-C (25.5%) and just a small number of children (n = 5) had high TG levels.

**Table 1:** Descriptive statistics for participants in the study

**Variable**	**Frequency (n)**	**Frequency (%)**
**Girls**	121	52.8
**Means of Transport**		
Walking	171	75
Cycling	0	0
Public transport	10	4.4
Car	43	18.8
**Socio Economic Status**		
Low	54	34
Medium	85	53
High	20	13
**Tanner Stage**		
2	32	15
3	87	39
4	78	35
5	25	11
	**Mean (SD)**	
**Age (years)**	11.65 (0.73)	
**% Body Fat**	22.92 (7.84)	
**Calories (Kcal)**	2133.95 (495.3)	
**Calories from Fat (Kcal)**	680.85 (374.06)	
**Waist Circumference (cm)**	70.71 (9.97)	
**Total Cholesterol (mg/dL)**	148.29 (26.39)	
**HDL-C (mg/dL)**	48.23 (13.31)	
**Triglycerides (mg/dL)**	69.69 (33.62)	
**Glucose (mg/dL)**	90.24 (7.83)	
**Average Systolic Blood Pressure (mm hg)**	116.35 (13.12)	
**Average Diastolic Blood Pressure (mm hg)**	61.19 (8.28)	
**Physical activity (min/day MVPA)**	39.53 (20.26)	

Legend: *SD* standard deviation, *HDL-C* high density lipoprotein-cholesterol, *kcal* kilocalories; *cm* centimeters, *mg/dL* milligrams per deciliter, *mm hg* millimeter of mercury, *min/day in MVPA* minutes per day in moderate to vigorous physical activity.

**Table 2:** Distribution of the metabolic syndrome and related components

**Metabolic Risk Factors**	**At risk definition**	**Frequency (%)**
**Waist Circumference**	≥90^th^ percentile	16.4
**HDL-C**	<40 mg/dL	25.5
**Triglycerides**	≥150 mg/dL	2.5
**Glucose**	≥100 mg/dL	8.8
**Blood Pressure**	≥130/≥85 mm hg	15.1
**Metabolic Syndrome**	WC ≥ 90^th^ + 2 other	3
**Physical Activity**	<60 min/day in MVPA	85.6

Legend: *HDL-C* high density lipoprotein- cholesterol, *mg/dL* milligrams per deciliter, *mm hg* millimeter of mercury, *WC* waist circumference, *min/day in MVPA* minutes per day in moderate to vigorous physical activity.

Results from crude and adjusted binary logistic regressions between metabolic risk factors and walking are presented in Table 3.

**Table 3:** Association between walking and metabolic risk factors

**Dependent variable**	**Crude OR**	**95%CI**	**Adjusted OR**	**95%CI**
**Waist Circumference**^**a**^	**2.28**	1.06 - 4.95*	**2.64**^**b**^	1.63-6.01*
**HDL-C**^**a**^	**2.33**	1.15 - 4.71*	**2.14**^**c**^	1.01-4.52*
**Triglycerides**^**a**^	2.04	0.33 - 12.50	-	-
**Glucose**^**a**^	1.17	0.40 - 3.43	-	-
**Blood Pressure**^**a**^	1.79	0.82 - 3.91	-	-

Legend: *HDL-C* high density lipoprotein-cholesterol, *OR* odds ratio, *95*% *CI* 95 percent confidence interval.

a) Reference Category: At risk.

b) Adjusted for gender, % body fat, minutes in moderate-to-vigorous physical activity.

c) Adjusted for age and tanner stage.

Crude binary logistic regressions showed significant associations between walking and WC < 90^th^ percentile (OR = 2.28, 95%CI = 1.06 - 4.95) and HDL-C ≥ 40 mg/dL (OR = 2.17, 95%CI = 1.10-4.25). Adjusted analysis still suggests higher odds, in walkers, to have a better WC (OR = 2.64, 95%CI = 1.63-6.01) and HDL-C (OR = 2.14, 95%CI = 1.01-4.52) profiles than non-active commuters. No significant interactions were found by walking on any metabolic risk outcome (results not presented).

## Discussion

The aim of this cross-sectional study was to analyze the relationship between ACS and the metabolic risk factors for children defined by IDF. Findings suggest a significant association of walking with both WC and HDL-C providing implications for children health promotion. Unlike other studies we controlled for confounding by dietary fat intake and MVPA, which would better estimate the impact of walking on the metabolic risk factors.

Our results suggest a beneficial association between walking to/from school and WC, after controlling for MVPA. In line with our findings Rowe and colleagues [26] compared active and passive school commuters and found that children who actively commute to school have healthier waist adiposity, body weight, cholesterol sub-fractions, blood lipids and possibly inflammation. Also a longitudinal research focusing on cycling to school and weight found lower odds of being overweight in children who cycled to school [27]. Despite the heterogeneity of ACS definitions, a recent review on ACS health-related fitness in children and adolescents found an association between this active behaviour with a healthier body composition [9]. These findings could be especially significant for obese children that usually have less PA opportunities implying that walking to school may be a simple and effective strategy, that can be implemented almost everywhere, to help control and reduce overweight.

While the association between walking and fatness has been well investigated, limited evidence exists regarding other metabolic health parameters of children who walk to school. Despite data in adults provide strong evidence that people who are more physically active have higher HDL-C levels, results vary considerably depending on the characteristics of the exercise programs and the assessing methods. In children, thus evidence suggest a favorable lipoprotein profile related to higher habitual physical activity, and that increases in HDL-C and reductions in low density lipoprotein cholesterol may be possible with regular exercise, the dose–response relationship remains elusive [28]. Findings from our study, add information to current knowledge suggesting that walking to school may be of relevance for a better HDL-C profile. In line with our findings, in a meta-analysis from Kodama and collaborators [29] including 25 articles, regular aerobic exercise was modestly associated with clinically important elevation in HDL-C levels. In addition the exercise duration has been previously highlighted as the most important element of an exercise prescription [30]. In fact, it is assumed that a minimum exercise volume is needed to significantly increase HDL-C [30]. Although, a dose–response relationship between the amount of exercise performed and HDL-C has been suggested [30,31] research shows conflicting results. The exercise volumes suggested by previous studies varies between 20 minutes of exercise 3 to 4 times a week and 60 minutes of combined exercises at a maximum heart rate of 75%. Interestingly, in our study 86% of the students took less than 15 minutes to get to school, and only 13% took more than 15 minutes (data not shown), still our results suggest that walking to/from school predicts higher HDL-C levels. This can indicate that even small amounts of walking may play an important role in health. Despite the fact that 75% of our sample were active commuters, a large percentage (85%) were not sufficiently active to reach the recommended daily MVPA. As point biserial correlation didn’t show any association between means of transport to/from school and minutes in MVPA this may imply that walking could be of light intensities and therefore not enough to be considered in PA recommendations. In fact is likely that exercise-induced changes in HDL-C are the result of the interaction amongst each exercise component (intensity, frequency, duration of each exercise session and length of the exercise training period) however the relative contribution of each exercise component has not been established yet [32], particularly in children. Apparently, evidence point toward favourable changes to occur incrementally and reach statistical significance at approximately 7–10 miles per week or 1200 to 1600 kcal, what seems to fit in our sample [32]*.* We could also speculate that walking is just about the only MVPA children engage most days of the week and children who walk to/from school may spend less time in other forms of MVPA.

In contrast to our results, a longitudinal research in children showed no difference in CVD risk factors between passive travelers and walkers suggesting that self-selected intensities of walking were not enough to improve cardiovascular health when compared to cycling [8]. Nonetheless, in our data the inclusion of several confounders such as body fat percentage, minutes of MVPA and fat intake did not significantly altered our regression model, one may suggest that walking was an important factor for the main differences in HDL-C levels. We may also speculate that intensities achieved when walking to and from school may be relevant enough for good HDL-C levels.

The present study focus on walking in 10 to 12 year old children by contrast with the most prevalent research that commonly focuses on the effects of total PA or different types of training in metabolic risk factors. To our knowledge data from the present study are novel information regarding the association between walking and metabolic risk factors in Portuguese youths. The use of objective measures of PA and anthropometry are also important strengths of this study. Differently from other research we adjusted analysis for potential confounders such as diet, SES, and demographics as the risk of metabolic syndrome is strongly associated with sociodemographic and lifestyle patterns including adequate diet, healthy weight and regular PA [33].

This study has some limitations notable of comment. Our independent variable, means of transport to and from school, relies on self-reported information. The cross sectional design of our study did not allow us to determine the direction of the observed associations. Nonetheless our data suggests that any active commuting is associated with better health. Finally the lack of results in other metabolic factors may be due to the lack of statistical power to show differences as was the case for triglycerides and glucose. Further research to clarify the threshold needed in each exercise component to obtain metabolic benefits should be conducted in children who walk to school in order to study the effect of this behaviour in health.

Future investigation should include Global Position System (GPS) technology during walking and combine it with accelerometer data. Therefore, more reliable information on time travelled, distance, intensities and patterns of this specific behaviour could be obtained and it would decrease the bias of self-reporting methods.

Even though walking has decreased in recent years, it still is an affordable and easy way of getting high daily PA levels, without the costs associated with regular exercise programs. Walking should be consider a major key factor to increase PA levels [14] and reduce important health outcomes such as obesity [34]. Countries such as Denmark and Holland show that it is possible to maintain and improve high levels of active commuting and our data reinforces that even small amounts of this behavior has potential to be of clinical relevance for health.

## Conclusion

In summary, the findings from this study showed that walkers have higher odds of having a better WC and HDL-C profile than children who use passive means of transport to/from school, after adjusting for MVPA and diet. For that reason exertions to increase and maintain walking in children may be particularly relevant as it is likely to positively impact health and decrease metabolic and CVD.

## Abbreviations

HDL-C: High density lipoprotein cholesterol; CVD: Cardiovascular disease; ACS: Active commuting to/from School; PA: Physical activity; WC: Waist circumference; TC: Total cholesterol; TG: Triglyceride; GLU: Fasting glucose; IDF: International Diabetes Federation; mg/dL: Milligrams per deciliter; mm hg: Millimeter of mercury; MVPA: Moderate to vigorous physical activity; cm: Centimeters; BMI: Body mass index; SES: Socio economic status; SD: Standard deviation; kcal: Kilocalories; OR: Odds ratio; 95% CI: 95 percent confidence interval; GPS: Global Position System.

## Competing interests

The authors declare that they have no competing interests.

## Authors’ contributions

ANP and MPS conceived and coordinated the study. ANP conceptualized and drafted the paper after contribution to data acquisition, analysis and interpretation. JCR, EM, JM, MPS contribute to further development and critical revision of the manuscript. JCR made substantial contributions data acquisition. EM assisted in the statistical analysis. MPS and JM contributed with grant funding for the project. All authors approved the final version of the manuscript.
